# The importance of early diagnosis and treatment of incidental tension pneumothorax during robotic assisted laparoscopy for diaphragmatic endometriosis: a report of two cases

**DOI:** 10.52054/FVVO.13.1.010

**Published:** 2021-03-31

**Authors:** MD Ribeiro, T Freire, F Leite, E Werebe, R Cabrera Carranco, W Kondo William

**Affiliations:** Department of Gynaecological Surgery, Hospital São Luiz Morumbi Rede Dor, São Paulo, Brasil; Department of Gynaecological Surgery, Hospital Israelita Albert Einstein, São Paulo, Brasil; Department of Gynaecological Surgery, Vita Batel Hospital, Curitiba, Brazil

**Keywords:** diaphragmatic endometriosis, pneumothorax

## Abstract

We describe two cases of diaphragmatic endometriosis treated using the robotic assisted laparoscopic approach, in which an incidental tension pneumothorax occurred during the initial inspection and assessment of diaphragmatic lesions. We demonstrate the importance of early diagnosis of this complication and report successful resolution using the thoracic drainage technique. In case one, after the pneumoperitoneum was installed, during the cavity assessment and inspection, small endometriotic lesions were observed in the tendon portion of the diaphragmatic surface. We observed a sudden increase in maximum airway pressures and a reduction in tidal volume, associated with arterial hypotension and hemodynamic instability and bulging of the diaphragm, which led to the diagnosis of a tension pneumothorax. In case two, diaphragmatic endometriotic lesions were also observed after hepatic mobilisation and following visualisation of the endometriotic lesions, an abrupt decrease in the capnography values was observed, consistent with hypertensive pneumothorax. In both cases, even after deflation of the abdominal cavity, hemodynamic instability persisted. We treated both cases with thoracic drainage, which immediately normalised respiratory parameters and resulted in hemodynamic stabilisation, and the surgical procedures were continued. During laparoscopic procedures for the treatment of diaphragmatic endometriosis, the endometriotic lesions can behave as communication hole in the tendon portion of the diaphragmatic surface and the changes in ventilatory patterns and haemodynamic instability should alert the medical team to the development of an incidental tension pneumothorax. The early identification of this complication in both cases allowed rapid intervention for chest drainage and allowed the surgical procedure to continue.

## Introduction

Endometriosis is a disease characterised by a chronic oestrogen dependent inflammatory process that mainly affects the pelvic organs. Multiple theories have been postulated about its aetiology. One of the most accepted theories describes how the detached endometrial cells travel retrograde to the pelvic organs (Bulun et al., 2019). In the vast majority of cases, the disease is found in the pelvis (Andres et al., 2020). Extra-pelvic disease occurring in the upper abdomen including the diaphragm is rare and represents a diagnostic challenge. In these cases, robotic assisted laparoscopic surgery associated with video assisted thoracoscopy (VATS) is the best way of treatment (Wolthuis et al., 2003). We describe two cases of robotic assisted laparoscopic surgery for the treatment of diaphragmatic endometriosis that developed an incidental tension pneumothorax during the visualisation of the lesions. Both were quickly treated using chest drainage.

## Case 1

A 38 year old nulliparous patient with a BMI of 20 and ASA I classification was diagnosed with infiltrating pelvic endometriosis (ASMR:IV, ENZIAN A2 B2 C3 FI FO FU). She gave a history of chronic pelvic pain, infertility, and right sub- scapular pain that worsened in the menstrual period. Previous surgical history included a laparoscopic rectosigmoidectomy, bilateral ureterolysis, left excision of ovarian endometrioma, left nerve decompression and bladder nodule shaving performed in another hospital 1 year previous. Following the initial laparoscopic procedure she developed sepsis and a right pleural effusion which was drained. She remained hospitalised for 23 days with persistent symptoms very similar to those that led her to initially seek medical attention. She underwent further surgery for the treatment of infiltrative pelvic endometriosis six months later with shaving of a rectal nodule, left ovarian implant vaporisation and excision of posterior cul-de-sac endometriosis implants. During laparoscopic assessment of the abdominal cavity, lesions of endometriosis were found on the diaphragmatic surface, but were not treated due to the potential higher infection risk due to intestinal manipulation and prolonged surgical time. After 6 months of medical treatment with suppression of menstruation with GnRH analogues (goserelin 3.6 mg monthly subcutaneously) and add back hormone therapy (transdermal oestradiol 25 mg twice weekly) for six months, upper abdominal magnetic resonance imaging (MRI) was performed. This did not detect any endometriotic lesions in the diaphragm and she was booked for second look surgery.

**Figure 1:**
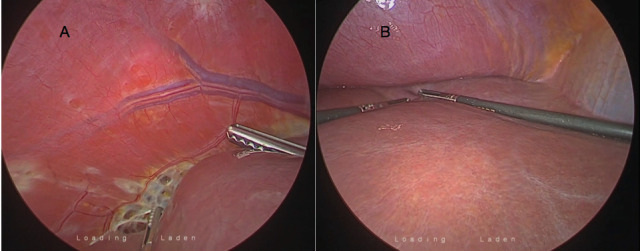
— A) Diaphragmatic fenestrations in the tendon portion of the direct diaphragm communicating with the thoracic cavity. B) Diaphragmatic dome bulging secondary to hypertensive pneumothorax, evident reversal sign of diaphragmatic concavity.

In the operating room, selective tracheal intubation was performed with a 35F double-lumen tube on the right, with no evidence of complications in the respiratory tract. The correct positioning of the double light tube was confirmed by pulmonary auscultation, showing effective selective ventilation. After proper positioning (standard position for pelvic and thoracic access) pneumoperitoneum was created with an initial intracavitary pressure of 15 mmHg. The cavity assessment was performed with the patient in the prone position, and after slight hepatic mobilisation, endometriotic implants were visualised on the right diaphragmatic surface (Figure 1A), compromising part of the central tendon in various forms of presentation. After moving to the Trendelenburg position, an assessment of the pelvic cavity was undertaken, where we identify fibrotic / endometriotic tissue as a result of previous surgery in the retrocervical region and the bottom of the vaginal sac. During the study of the pelvic cavity, approximately 18 minutes after the start of surgery, an increase in maximum airway pressure was observed, reaching 35 mmHg, with a reduction in tidal volume and arterial hypotension (> 20% of basal pressure) maintaining adequate oxygen saturation. After the alert from the anesthesiologist, the surgical team identified bulging of the diaphragm (Figure 1B) and visualised small communication holes in the endometriotic lesions in the right diaphragm, which after correct mobilization of the liver for visualisation, served as a mechanism for the formation of hypertensive pneumothorax. In view of the clinical evidence, the CO_2_ insufflator was switched off and a chest drain was placed in the fourth right intercostal space, mid-axillary line. After drainage, there was an immediate normalisation of the maximum airway pressures and hemodynamic stabilisation. With effective drainage, the abdominal cavity was insufflated with 15 mmhg and surgical treatment of the respective lesions was continued, assisted by robotic laparoscopy. To treat diaphragmatic lesions, monopulmonary ventilation was installed and video-assisted thoracoscopy was performed to aid in resection of the lesions, delimitation of the surgical margin, and identification of important anatomical structures (vena cava, supra-hepatic, hiatus, and nerves) for proper management and reconstruction of the diaphragmatic defect. Using a laparoscopic approach, the right falciform and triangular ligaments were dissected, with resection of a multi-fenestrated endometriotic lesion in the central tendon of the right diaphragm measuring approximately 8 cm, followed by reconstruction using robotic assisted laparoscopic suture. The surgery time was 200 minutes.

## Case 2

**Figure 2:**
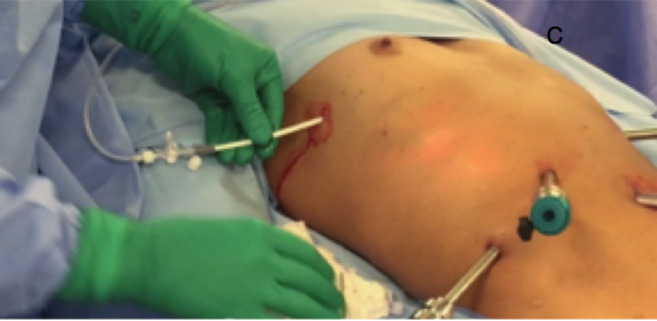
— Thoracic drainage in case 1.

A 45-year-old nulliparous patient with a BMI of 25.6 and ASA grade II presented to the department with a diagnosis of deep endometriosis (ASMR IV ENZIAN :A3 B2 C3 FO FU FI). She was booked for a surgical treatment of pelvic and diaphragmatic endometriosis following suppression of menstruation with GnRH analogues. The planned surgery included a robotic laparoscopic resection of diaphragmatic lesions assisted by video- thoracoscopy. While pelvic inspection was being performed, an abrupt change in capnography (CO_2_) was noted, concomitantly with arterial hypotension, maintaining adequate saturation of oxygen. With direct laparoscopic vision, multiple holes were found in the diaphragm in the endometriotic lesions, which acted as communicating lesions during hepatic mobilisation. As in the previous case immediate desufflation of the abdominal cavity and treatment of the tension pneumothorax were performed (puncture in the second intercostal space with a 14F venous catheter to decompress the lung) (Figure 2). After drainage, capnography normalised immediately, with hemodynamic stabilisation. The surgery was uneventful, with an approach similar to case I, with robotic assisted laparoscopy and video-assisted thoracoscopy to treat the diaphragmatic disease. The patient was extubated in the operating room, without respiratory difficulties, and thoracic drainage was placed and maintained until the second postoperative day.

## Discussion

The incidence of diaphragmatic endometriosis is uncommon and difficult to diagnose (Andres et al., 2020). In most cases, it represents an incidental finding, with lesions observed by laparoscopy generally behind the right lobe of the liver (Ceccaroni et al., 2012). Compatible with the literature, in both reported cases, the MRI examinations did not show the lesions.

Diaphragmatic fenestrations in endometriotic tissue are described in the literature as a mechanism of pneumothorax formation, being responsible for 7.3-36% of cases of spontaneous catamenial pneumothorax (Gil et al., 2020). Although it occurs on the right side in approximately 90% of cases, it can be on the left or bilateral (Narula et al., 2018). Pelvic endometriosis is associated in 30- 51% of the cases (Visouli et al., 2012; Segawa et al., 2011). Spontaneous pneumothorax formation in these patients is due to the transdiaphragmatic passage of air through the peritoneal cavity and its air, from the cervix during the menstrual period (Frost et al., 2012). However, the opening of diaphragmatic fenestrations in endometriotic tissue during pneumoperitoneum has not yet been described in the investigated literature (Ceccaroni et al., 2012). Video-laparoscopic treatment can lead to the incidental opening of small holes in the diaphragmatic endometrial tissue during surgical manipulation, even without direct tissue trauma, causing incidental pneumothorax. Intraoperative hypertensive pneumothorax is a serious event, with hemodynamic and respiratory repercussions, requiring immediate diagnosis and treatment The progressive increase in intrathoracic pressure, as a result of air trapping between the pleura, causes collapse of the affected lung and deviation of the mediastinum to the contralateral side, which interferes with venous return and ventilation (Zugliani et al., 2008).

In both cases presented, the simple movement of the liver to visualise the lesions, associated with the maintenance pressure of the pneumoperitoneum, was enough to open communication channels in the endometrial tissue and the formation of tension pneumothorax, even without surgical manipulation of the endometriosis lesions. Diaphragmatic endometriotic tissue remains a possible route of communication with the chest, and with increasing pressures in the abdominal cavity, suspected pneumothorax should be considered with clinical signs suggest this. Capnography indirectly reflects the state of pulmonary circulation and CO_2_ supply to the right heart chambers. Low cardiac output (shock) reduces perfusion of the alveolar segments, which therefore do not participate in gas exchange. Furthermore, collapse of the lung areas due to pneumothorax can lead to an abrupt decrease in EtC02 values on capnography, which is a warning sign found in diaphragmatic endometriotic resections. In the second reported case, no significant increase in airway pressure was detected, but the reduction in EtC0_2_ values served as a parameter for diagnostic suspicion. In a case report of pneumothorax during anaesthesia for puncture of an ultrasound-guided breast tumor, reduction of EtCO_2_ was also one of the first indicative signs of pneumothorax (Oliveira et al., 2004). Such conditions should be considered, since prompt diagnosis and treatment are necessary to avoid serious repercussions and even death. In both cases, thoracic drainage was chosen, which caused immediate normalisation of respiratory parameters and hemodynamic stabilisation, allowing surgery to continue.

## Conclusion

Intraoperative pneumothorax is a complication that can occur in patients with pelvic endometriosis treated with any kind of minimally invasive approach as diaphragmatic endometriosis is often under-diagnosed by imaging tests. The anesthesiologist and the surgeon should be aware that such a complication is a possibility for these patients and should be prepared to diagnose and treat this complication immediately.
